# Quality assessment of the wide-angle detection option planned at the high-intensity/extended *Q*-range SANS diffractometer KWS-2 combining experiments and *McStas* simulations

**DOI:** 10.1107/S160057672400493X

**Published:** 2024-06-27

**Authors:** Aurel Radulescu

**Affiliations:** a Jülich Centre for Neutron Science at Heinz Maier-Leibnitz Zentrum (MLZ) Forschungszentrum Jülich Lichtenbergstraße 1 Garching 85747 Germany; Argonne National Laboratory, USA

**Keywords:** small-angle neutron scattering, SANS, wide-angle neutron scattering, WANS, *McStas* simulations, semi-crystalline materials

## Abstract

For a reliable characterization of materials and systems featuring multiple structural levels, a broad length scale from a few ångström to hundreds of nanometres must be analyzed and an extended *Q* range must be covered in X-ray and neutron scattering experiments. For certain samples or effects, it is advantageous to perform such characterization with a single instrument. Neutrons offer the unique advantage of contrast variation and matching by D-labeling, which is of great value in the characterization of natural or synthetic polymers. Some time-of-flight small-angle neutron scattering (TOF-SANS) instruments at neutron spallation sources can cover an extended *Q* range by using a broad wavelength band and a multitude of detectors. The detectors are arranged to cover a wide range of scattering angles with a resolution that allows both large-scale morphology and crystalline structure to be resolved simultaneously. However, for such analyses, the SANS instruments at steady-state sources operating in conventional monochromatic pinhole mode rely on additional wide-angle neutron scattering (WANS) detectors. The resolution must be tuned via a system of choppers and a TOF data acquisition option to reliably measure the atomic to mesoscale structures. The KWS-2 SANS diffractometer at Jülich Centre for Neutron Science allows the exploration of a wide *Q* range using conventional pinhole and lens focusing modes and an adjustable resolution Δλ/λ between 2 and 20%. This is achieved through the use of a versatile mechanical velocity selector combined with a variable slit opening and rotation frequency chopper. The installation of WANS detectors planned on the instrument required a detailed analysis of the quality of the data measured over a wide angular range with variable resolution. This article presents an assessment of the WANS performance by comparison with a *McStas* [Willendrup, Farhi & Lefmann (2004). *Physica B*, **350**, E735–E737] simulation of ideal experimental conditions at the instrument.

## Abstract 2

The performance of the wide-angle neutron scattering option on pinhole small-angle scattering instruments to measure data over a wide angular range with variable resolution can be assessed by comparison with the *McStas* simulation of ideal experimental conditions on the instrument.

## 1. Introduction

Materials based on semi-crystalline polymers exhibit a phase separation in crystalline and amorphous regions. These materials are characterized by a complex morphology with multiple hierarchically organized structural levels that span a wide length scale between several ångströms and hundreds of nanometres (Akpalu, 2010 ▸; Radulescu *et al.*, 2015*a* ▸; Kanaya *et al.*, 2007 ▸). Moreover, the bulk and interlamellar amorphous regions can be functionalized, which results in even more complex morphologies when external stimuli such as humidity, temperature or elongation/compression are applied to the sample (Schiavone *et al.*, 2023 ▸). For the characterization of such complex morphologies, a wide length scale has to be covered, which usually requires a combination of different experimental methods in the structural analysis. Combination of wide- and small-angle scattering techniques with X-rays (WAXS and SAXS) has long been used in such experimental investigations (Kanaya *et al.*, 2007 ▸; Koga *et al.*, 2008 ▸; Hama & Tashiro, 2003 ▸; Schneider, 2010 ▸). SAXS provides information about the overall morphology of polymers and their higher-order structural levels, while the crystalline structure of polymers can be investigated with WAXS. Similarly to X-rays, neutrons are an excellent probe for characterizing such complex morphologies over a wide length scale. Additionally, neutrons offer the unique advantage of different interactions with the ^1^H and ^2^H (deuterium, D) hydrogen isotopes, thus offering the possibility to vary the scattering contrasts between different constituents of a hydro­carbon sample over a broad range by H/D substitution (Jacques & Trewhella, 2010 ▸). This makes the small-angle (SANS) and wide-angle (WANS) neutron scattering techniques particularly suitable for the detailed investigation of natural and synthetic multi-component polymeric materials. However, while modern X-ray laboratory diffractometers or beamlines at large-scale facilities can easily and successfully combine ultra-small-angle X-ray scattering (USAXS), SAXS and WAXS (Pauw *et al.*, 2021 ▸), in the case of neutron scattering such a combination of methods requires special care. WANS and SANS can be simultaneously performed without technical or organizational difficulties with time-of-flight (TOF) SANS instruments at spallation sources. In this case, a broad wavelength band is used and a wide angular range of scattering can be covered by using a large number of detectors, either movable (Heenan *et al.*, 2005 ▸; Zhao *et al.*, 2010 ▸) or placed in fixed positions (Takata *et al.*, 2014 ▸; Koizumi *et al.*, 2020 ▸; Allen, 2023 ▸). In contrast, classical pinhole SANS diffractometers at steady-state neutron sources (nuclear reactors) must be combined with wide-angle neutron diffractometers to extend the *Q* range to higher *Q* values. However, simultaneous use of wide- and small-angle scattering methods in the same experiment is necessary for sensitive or expensive samples, if special attention must be paid to sample preparation (composition, quality, amount *etc*.) or *in situ* treatment (temperature, humidity, chemical condition *etc*.) during the experimental investigation (Tashiro & Sasaki, 2003 ▸). To overcome these difficulties, one should thus be able to cover an extended *Q* range at the same pinhole SANS diffractometer. Some pinhole instruments have already been equipped with focusing lenses for measurements at lower *Q* values (Koizumi *et al.*, 2007 ▸; Wood *et al.*, 2018 ▸; Barker *et al.*, 2022 ▸) and with wide-angle detectors for covering higher *Q* values (Heller *et al.*, 2018 ▸) in an attempt to extend their *Q* range as far as possible in both directions.

The small-angle neutron diffractometer KWS-2 (Radulescu *et al.*, 2012*a* ▸), operated by the Jülich Centre for Neutron Science at the Heinz Maier-Leibnitz Zentrum (MLZ), Garching, Germany, is dedicated to the investigation of mesoscopic multi-scale structures and structural changes in soft condensed matter and biophysical systems. Following demands from the user community, it was repeatedly upgraded to enable the exploration of a broad *Q* range, between 2.0 × 10^−4^ and 1.0 Å^−1^, providing high neutron intensities and a tunable experimental resolution (Radulescu *et al.*, 2015*b* ▸; Houston *et al.*, 2018 ▸). The broad *Q* range is currently covered by combining in a versatile user-friendly approach the conventional pinhole and focusing working modes. In the conventional pinhole mode, the wavelength λ and the sample-to-main-detector distance *L*_D_ can be varied between 2.8 and 20 Å and from 1.25 to 20 m, respectively, to maximize the *Q* range. The main detection system of KWS-2 consists of an array of ^3^He tubes and rapid read-out electronics (Houston *et al.*, 2018 ▸). In focusing mode, parabolic MgF_2_ lenses are used in combination with a small aperture at the entrance of the collimation system and a secondary scintillation-type high-resolution detector (HRD) that can be automatically brought in-beam on demand (Radulescu *et al.*, 2012*a* ▸).

With the ultimate goal of providing KWS-2 with diffraction capabilities that bridge from atomic- to meso-scale structures, a new upgrade of the instrument is currently underway. The aim is to combine short wavelengths (λ = 2.8 Å) with wide-angle detection of scattered neutrons up to a scattering angle θ_s_ = 50° to achieve a *Q*_max_ = 2.0 Å^−1^. This allows a broad *Q* range between 2 × 10^−4^ and 2.0 Å^−1^ to be covered, enabling full structural characterization of the sample of interest using only one instrument and one sample geometry. The current upgrade will then allow WANS and SANS measurements on KWS-2 in different combinations of standard and TOF modes with adjustable resolution for different scientific objectives: observation of crystalline peaks or form factor details of small-size morphologies, minimization of incoherent background, *etc*. Recent studies (Schiavone *et al.*, 2023 ▸; Hirai *et al.*, 2019 ▸) of multi-component systems characterized by different structural levels have investigated both the meso and short length scales in one experiment. These studies show the tremendous gain in structural information that can be achieved when a broad *Q* range can be covered in a combined SANS–WANS experimental approach at the same instrument. In addition, the WANS detectors at KWS-2 will enable suitable TOF conditions to gain insight into the inelastic scattering of hydrogenated samples (Balacescu *et al.*, 2021 ▸) and reduce the incoherent background, which is useful when data collected at high *Q* do not show a flat profile.

The two future detection systems at KWS-2, the SANS and WANS detectors, will operate independently, with the collimation, resolution and TOF conditions set for defined scientific purposes. The data quality of the main SANS detector has been characterized in detail in a previous publication (Houston *et al.*, 2018 ▸). The results of recent tests on KWS-2 to assess the quality of the measured data with adjustable resolution at different scattering angles covering a wide angular range beyond that of the conventional SANS setup are reported here. They are compared with the results of *McStas* (Willendrup *et al.*, 2004 ▸) simulations of the virtual ideal experimental setup.

## 2. Experimental

In this study, the reflection from the crystalline planes (111) of fullerene-C60 was followed for the assessment of the data quality measured at KWS-2 in TOF mode in both the SANS and WANS regimes. Fullerene-C60 powder from Sigma–Aldrich (Germany) was first structurally characterized by X-ray diffraction (XRD) in the range of θ_s_ between 5 and 60° with a Bruker 2nd Gen-D2 Phaser X-ray powder diffractometer (Cu source), and then prepared in a Hellma banjo-type quartz cuvette for investigation at the pinhole SANS diffractometer KWS-2 (Radulescu *et al.*, 2015*b* ▸; Houston *et al.*, 2018 ▸). XRD characterization of a silver behenate (AgBeh) powder sample (AlfaAesar, Germany) was carried out in parallel.

SANS was performed in the high-*Q* regime using the main detector at *L*_D_ = 1.25 m for two experimental setups: (i) with a wavelength λ = 3.0 Å as provided by a 36-blade velocity selector (Airbus, Germany) used in a tilted position in the beam (tilt angle ξ = −10°) for a wavelength resolution of Δλ/λ = 22.7% (Houston *et al.*, 2018 ▸), and (ii) with a wavelength λ = 2.8 Å with Δλ/λ = 4.7% in TOF mode with the resolution chopper (Radulescu *et al.*, 2015*b* ▸) used together with a tilted 72-blade velocity selector (Airbus, Germany) in a tilted position in the beam (ξ = −10°). The two velocity selectors provide a nominal full wavelength distribution Δλ/λ of 20.6% and 10.9%, respectively, when they are used in the standard configuration (λ_min_ = 4.5 Å), parallel to the beam axis, and of 22.7% (λ_min_ = 3.0 Å) and 14% (λ_min_ = 2.8 Å), respectively, when they are used in the tilted position at the maximum rotation speed (Radulescu *et al.*, 2023 ▸; Houston *et al.*, 2018 ▸). The weaker worsening of wavelength resolution in the inclined configuration for these short wavelengths compared with λ 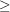 4.5 Å [35.3% and 18.5%, respectively, as reported by Houston *et al.* (2018 ▸) and Radulescu *et al.* (2023 ▸)] is due to the effect induced by the neutron guide cut-off of the KWS-2 instrument (Radulescu *et al.*, 2012*b* ▸, Houston *et al.*, 2018 ▸). The cut-off of a neutron guide refers to the rapid drop in brightness at lower wavelengths caused by the upstream guide’s curvature combined with the wavelength dependence of the critical angle of reflection from the mirror coating of the guide. The guide cut-off is also responsible for the slightly different λ_min_ obtained when the two selectors are used at maximum rotation speed in the tilted configuration. The opening of the rectangular source aperture, which was located in front of the sample at a collimation length of *L*_C_ = 4 m, was 50 × 50 mm, while the opening of the sample aperture was 10 × 10 mm. The data were collected on the main detector of the instrument, which consists of an array of ^3^He tubes with a pixel size of 8 × 8 mm.

The WANS test investigation was carried out on the fullerene-C60 sample with a portable scintillation HRD installed at the sample position, immediately after the sample (*L*_D_ = 30 cm), at an angle of 30° between the detector axis and the beam axis. The active area of the HRD is characterized by a diameter of 9 cm and the pixel size is 0.4 × 0.4 mm. Data were collected with λ = 5 Å, delivered by the tilted velocity selector. A resolution of Δλ/λ = 6.2% was obtained by collecting data in TOF mode using the chopper in concert with the velocity selector (Radulescu *et al.*, 2015*b* ▸).

Typical corrections for the empty cuvette contribution, dark current and detector sensitivity determined with a slab of Plexiglas secondary standard (Houston *et al.*, 2018 ▸) were applied to the raw data. The SANS data were subsequently calibrated in absolute units using the Plexiglas secondary standard (Houston *et al.*, 2018 ▸) and radially averaged over the entire detection area, while the WANS data were averaged only on a narrow equatorial sector of the HRD.

## 3. Simulations

Data measured on fullerene-C60 were compared with the results of simulations of the ideal experimental conditions, including neutron source, guide system, selector, collimator, sample and detector, using the *McStas* program package (Willendrup *et al.*, 2004 ▸). The KWS-2 instrument option for *McStas*, including different velocity selector states, was used in previous simulations as reported by Radulescu and co-workers (Radulescu *et al.*, 2012*b* ▸; Radulescu & Ioffe, 2008) ▸. For the currently reported simulation, the *McStas* component *Powder1.comp* was used to describe the fullerene-C60 powder sample, on the basis of the scattering pattern observed by XRD (Sanz *et al.*, 2015 ▸).

## 4. Results and comments

AgBeh is a wavelength calibrant used in SAXS and SANS, as reported by Ilavsky *et al.* (2018 ▸) and Houston *et al.* (2018 ▸). The Bragg spacing of AgBeh is known to be *d*_AgBeh_ = 58.38 Å (Okabe *et al.*, 2007 ▸). However, the crystalline scattering pattern of AgBeh in the wide-angle scattering regime, up to *Q*_max_ = 2.0 Å^−1^, is characterized by a large number of peaks (Tan *et al.*, 2020 ▸) that make it difficult to use this system for the purpose of calibration in wide-angle measurement geometry. Optionally, NIST X-ray powder diffraction calibration standards such as SRM 640 based on silicon and SRM 660 based on lanthanum hexaboride, LaB_6_, are available for the wide-angle (diffraction) scattering regime. These are routinely used at desktop and synchrotron devices such as the USAXS/SAXS/WAXS instrument at the APS, Argonne National Laboratory, USA (Ilavsky *et al.*, 2018 ▸).

**Figure 1:**
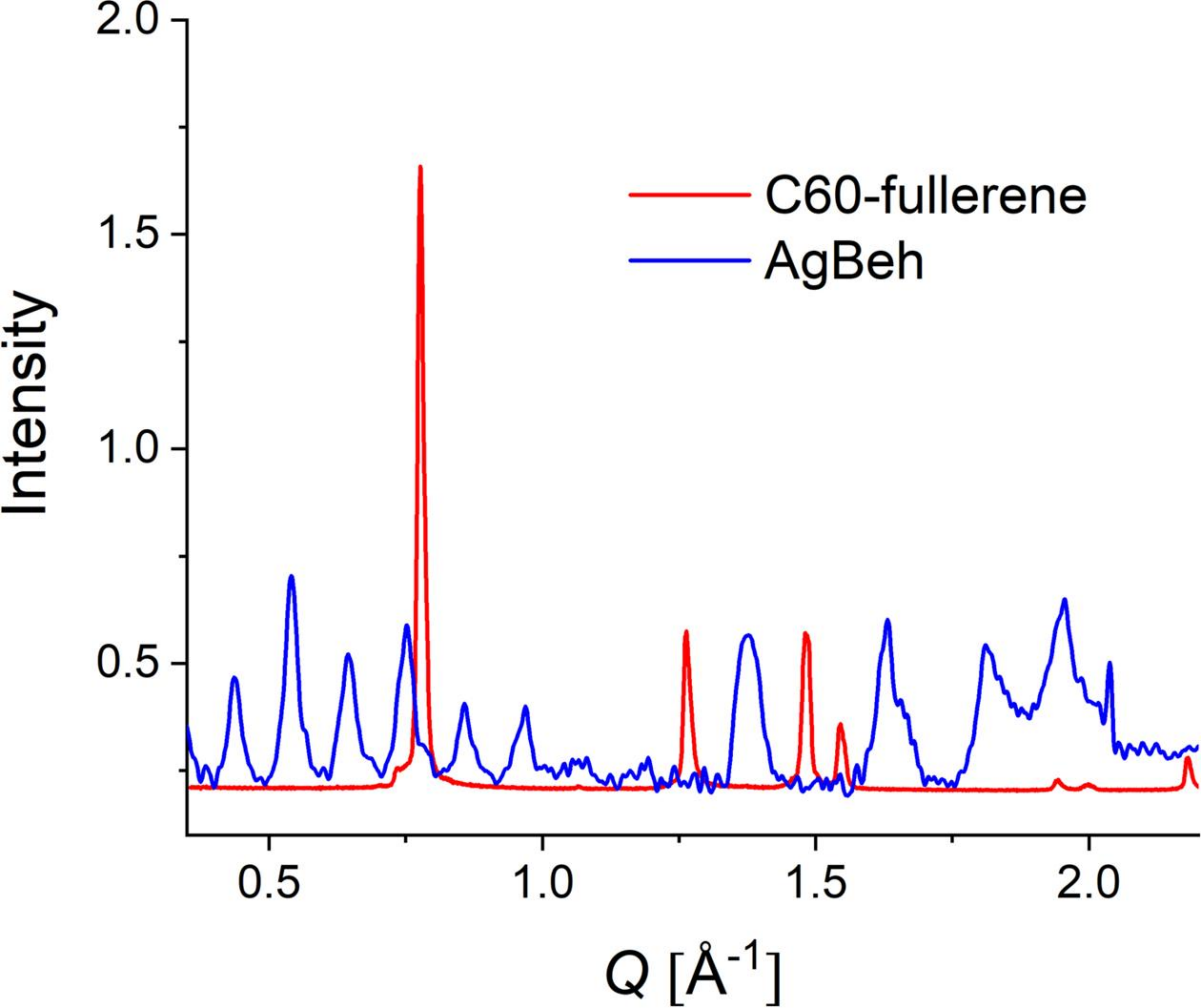
Scattering patterns from silver behenate (AgBeh) and C60-fullerene obtained by XRD.

In contrast to AgBeh, fullerene-C60 is characterized by isolated and very sharp crystalline reflections in the *Q* range corresponding to wide-angle scattering. The reflection from the crystalline planes (111) appears at θ_s_ = 10.78° in XRD patterns obtained with a Cu *K*α source (Sathish & Miyazawa, 2010 ▸). In this *Q* range, this crystalline reflection will appear isolated at *Q*_C60_ = 0.766 Å^−1^ (Sanz *et al.*, 2015 ▸). Fig. 1 ▸ shows the XRD results from the fullerene-C60 sample in parallel with those measured on AgBeh. The suitability of using the fullerene-C60 powder sample as a model system and observing the 111 reflection for the purpose of assessing the data quality measured in the WANS regime is evident from this comparison. In a SANS experiment, this reflection will be observed as a ring appearing at the scattering angles of θ_s_ = 19.68° when neutrons with λ = 2.8 Å are used, while with neutrons with λ = 5.0 and 7.0 Å the peak will be observed in the WANS regime at θ_s_ = 35.53 and 50.57°, respectively.

**Figure 2:**
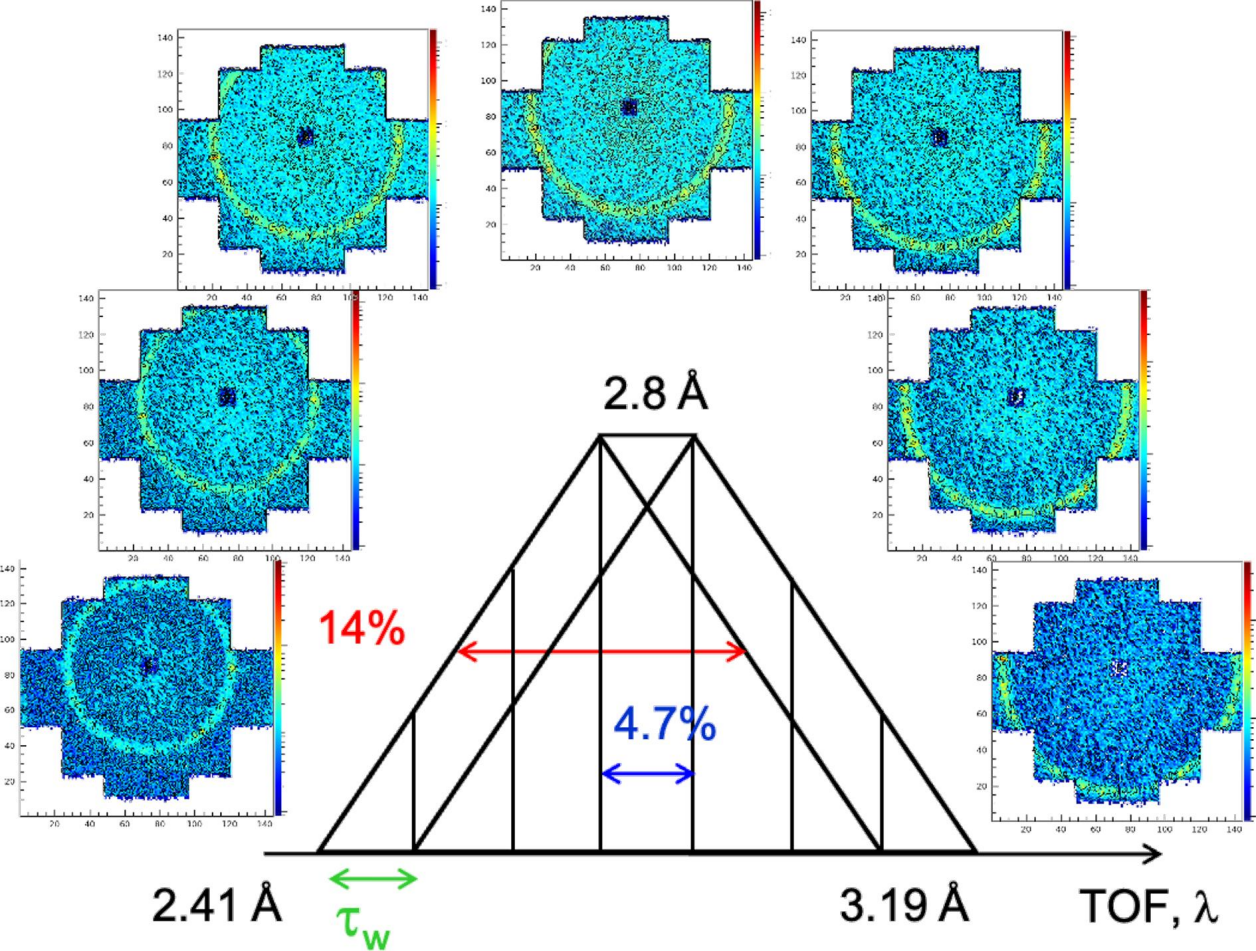
Scattering patterns from the fullerene-C60 powder sample obtained by TOF-SANS at KWS-2 for the following experimental conditions: λ = 2.8 Å and Δλ/λ = 14%, as delivered by the tilted velocity selector, *L*_D_ = 1.25 m, *f*_chopper_ = 98.24 Hz, Δφ = 25.72°, τ_w_ = 0.00072 s, Δλ/λ_aim_ = 4.7% (central TOF channel), number of channels *n*_chan_ = 7.

Fig. 2 ▸ shows the splitting scheme of the trapezoidal wavelength distribution acquired in TOF mode for a pulse delivered by the chopper when measuring the 111 reflection from the fullerene-C60 powder sample. The wavelength delivered by the velocity selector was λ = 2.8 Å. The scattering patterns corresponding to each time channel as recorded on the main ^3^He detector are shown. The double-disc resolution chopper pulsed the beam and transformed the triangular distribution delivered by the velocity selector with Δλ/λ = 14% into a trapezoidal one, due to the opening time of the chopper window, τ_w_. This is because the KWS-2 double-disc chopper can practically be considered as a single-disc chopper, since the two discs rotate at the same speed (Radulescu *et al.*, 2015*b* ▸). In this case, the TOF resolution is determined by τ_w_, which depends on the chopper frequency *f*_chopper_ and the angular opening of the window 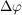. 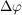 can be adjusted by suitable positioning of the two discs relative to each other. Technical details and setting parameters of the KWS-2 chopper are reported by Radulescu *et al.* (2015*b* ▸). In the simple TOF mode with the KWS-2 chopper, one aims to divide the wavelength band into *n* equal parts (as shown schematically in Fig. 2 ▸). In principle, τ_w_ is set so that it corresponds to the *n*th part of the wavelength band. For the SANS measurement of the fullerene-C60 powder sample *f*_chopper_ and Δφ were set to deliver TOF channels with a width of τ_w_ = 0.00072 s, which allowed the trapezoidal pulse delivered by the selector/chopper tandem to be split into seven TOF channels. Thus, the width of the central TOF channel corresponded to Δλ/λ = 4.7%. The TOF–distance plots and examples of different experimental setups to achieve the desired resolution in the TOF channels of the split wavelength distribution are described in detail by Radulescu *et al.* (2015*b* ▸).

The 2D scattering pattern corresponding to each TOF channel displays the narrow ring-like scattering feature from the 111 reflection of the powder sample. The position of the ring scattering feature on the 2D detector changes from channel to channel because of the different wavelength corresponding to each channel, which makes the scattering from the same structural feature to appear at a different scattering angles (Radulescu *et al.*, 2015*b* ▸).

**Figure 3:**
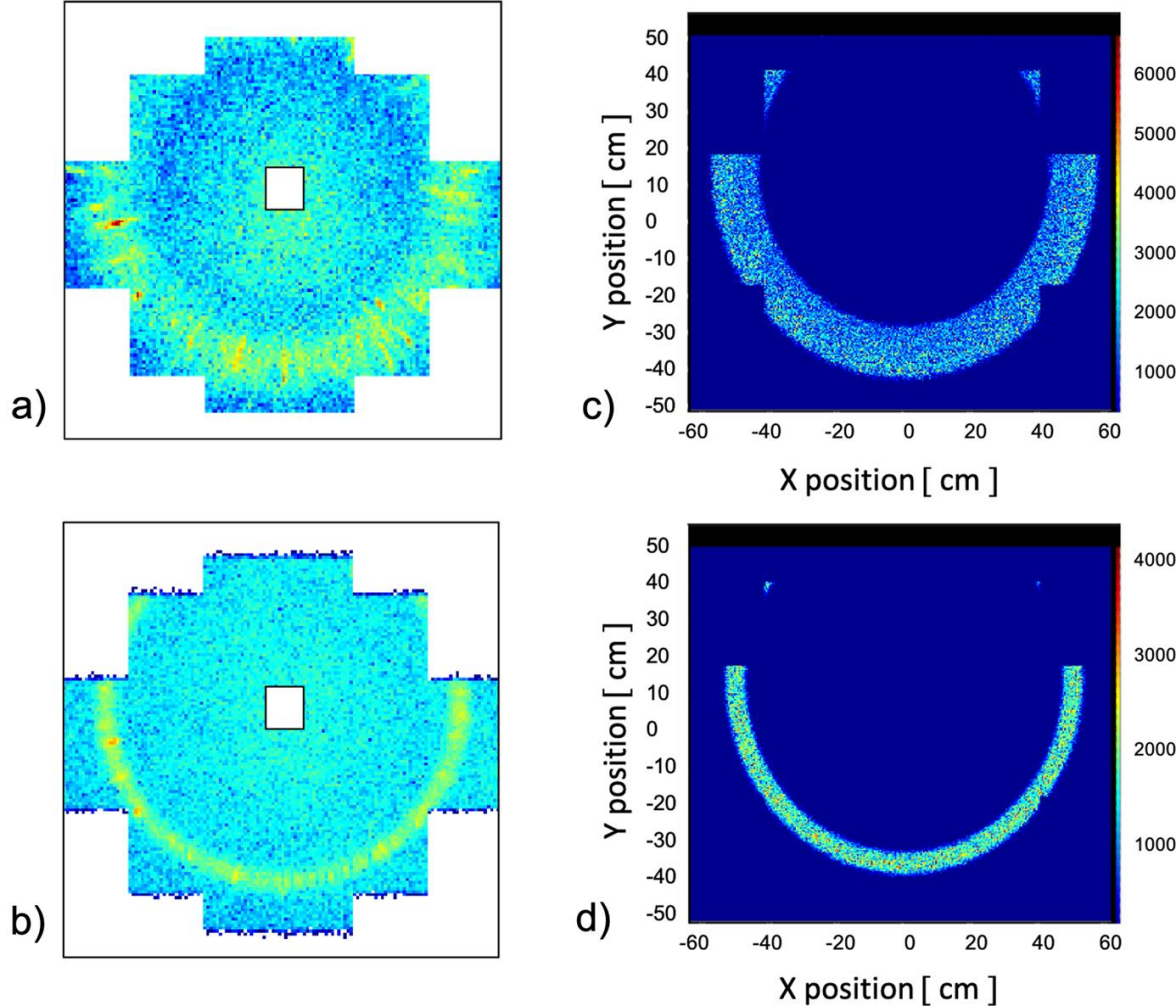
Measured (*a*, *b*) and simulated (*c*, *d*) two-dimensional scattering patterns from the fullerene-C60 powder sample for λ = 3.0 Å and Δλ/λ = 22.7% (*a*, *c*) and λ = 2.8 Å and Δλ/λ = 4.7% (*b*, *d*).

Fig. 3 ▸ presents a comparison of the 2D SANS patterns from the fullerene-C60 sample as delivered by measurements with and without the chopper and the *McStas* simulations for the same conditions, namely λ = 3.0 Å with Δλ/λ = 22.7% when only the velocity selector is considered [panels (*a*) and (*c*), respectively] and λ = 2.8 Å with Δλ/λ = 4.7% [panels (*b*) and (*d*), respectively]. A broad ring-like scattering feature representing the 111 reflection from the fullerene-C60 powder sample can be observed towards the rim of the detector in both experimental and simulated cases for the conventional measurement mode. Meanwhile, as a consequence of a better wavelength resolution, a very narrow width ring-like scattering feature is observed in both measured and simulated scattering patterns when the chopper is used in concert with the velocity selector and the data are sorted in TOF mode. Fig. 3 ▸(*b*) presents the scattering pattern corresponding only to the central TOF channel (the 4th TOF channel in the trapezoidal distribution shown in Fig. 2 ▸), characterized by λ = 2.8 Å and Δλ/λ = 4.7%. The large width of the scattering feature measured in the conventional mode is due to the large Δλ/λ used in this experiment, while the asymmetric aspect of the scattering pattern with respect to the detector geometry is due to the off-centered positioning of the detector in the beam. Although this is outside the scope of the present study and no additional analysis was performed here, it can be speculated that the fringe features observed along the azimuthal and radial directions in Fig. 3 ▸(*a*) could be an effect of the granular powder character of the sample (Kaduk *et al.*, 2021 ▸), which was investigated under the broad Δλ/λ measurement conditions.

**Figure 4:**
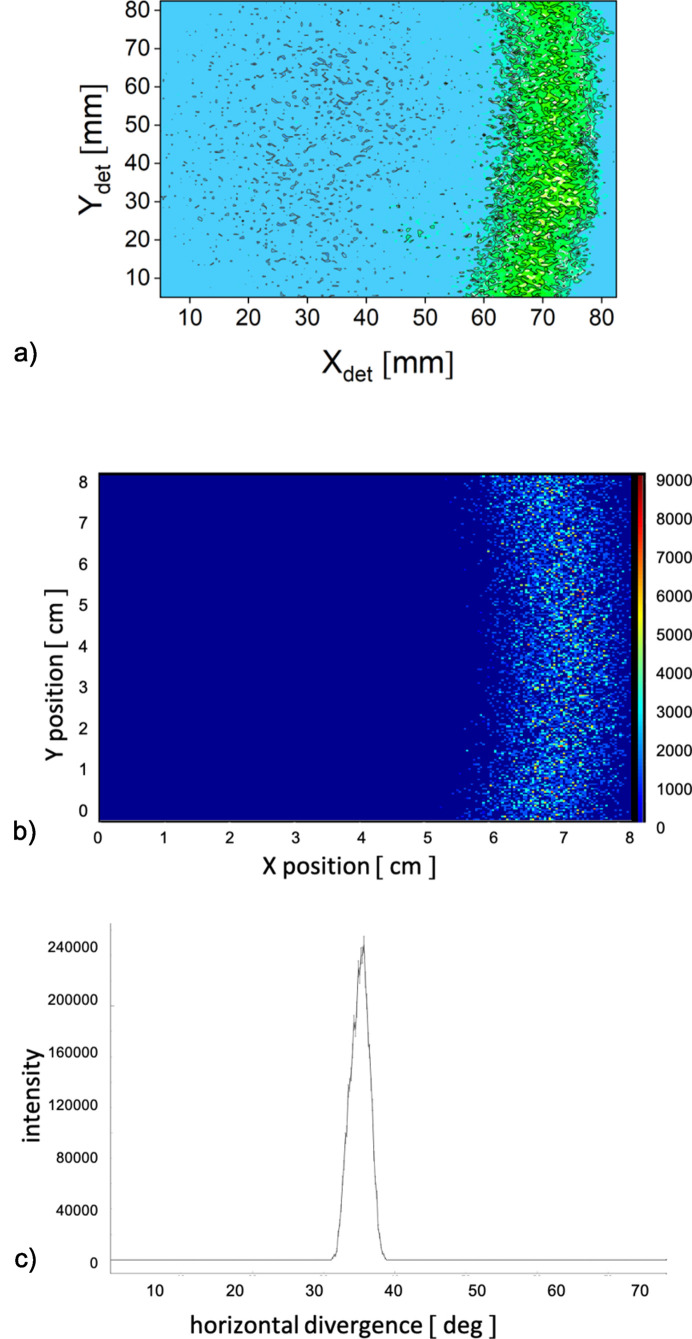
Measured with an HRD (*a*) and simulated (*b*) two-dimensional scattering patterns from the fullerene-C60 powder sample for λ = 5.0 Å and Δλ/λ = 6.2%. The HRD was used in the TOF-WANS mode (see text). Panel (*c*) shows the simulated data as a function of the scattering angle using the *Div1D_monitor McStas* component.

The scattering results from the 111 reflection of the fullerene-C60 powder sample collected in TOF mode with the portable HRD installed at the sample position in WANS geometry are shown in Fig. 4 ▸(*a*). Again only results from the central TOF channel of the wide trapezoidal distribution provided by the velocity selector/chopper tandem are displayed. The experiment was carried out with a neutron wavelength λ = 5 Å, as delivered by the velocity selector at a wavelength spread of Δλ/λ = 18.5%. The chopper settings were adjusted to provide a central TOF channel width corresponding to Δλ/λ = 6.2%. This allowed us to split the pulse delivered by the selector/chopper tandem into seven TOF channels, similarly to the scheme shown in Fig. 2 ▸. Figs. 4 ▸(*b*) and 4 ▸(*c*) show the simulated results for the same experimental conditions as a function of *x*, *y* position on the 2D detector and of scattering angle using the *PSD_monitor* and *Div1D_monitorMcStas* components, respectively. Arcs of high intensity were observed on the HRD, representing a portion of the ring-like scattering feature due to the 111 reflection from the powder sample. For these experimental conditions, the 111 reflection is observed at θ_s_ = 35.53°, out of the center of the HRD, which corresponds to an angle of θ_s_ = 30°.

**Figure 5:**
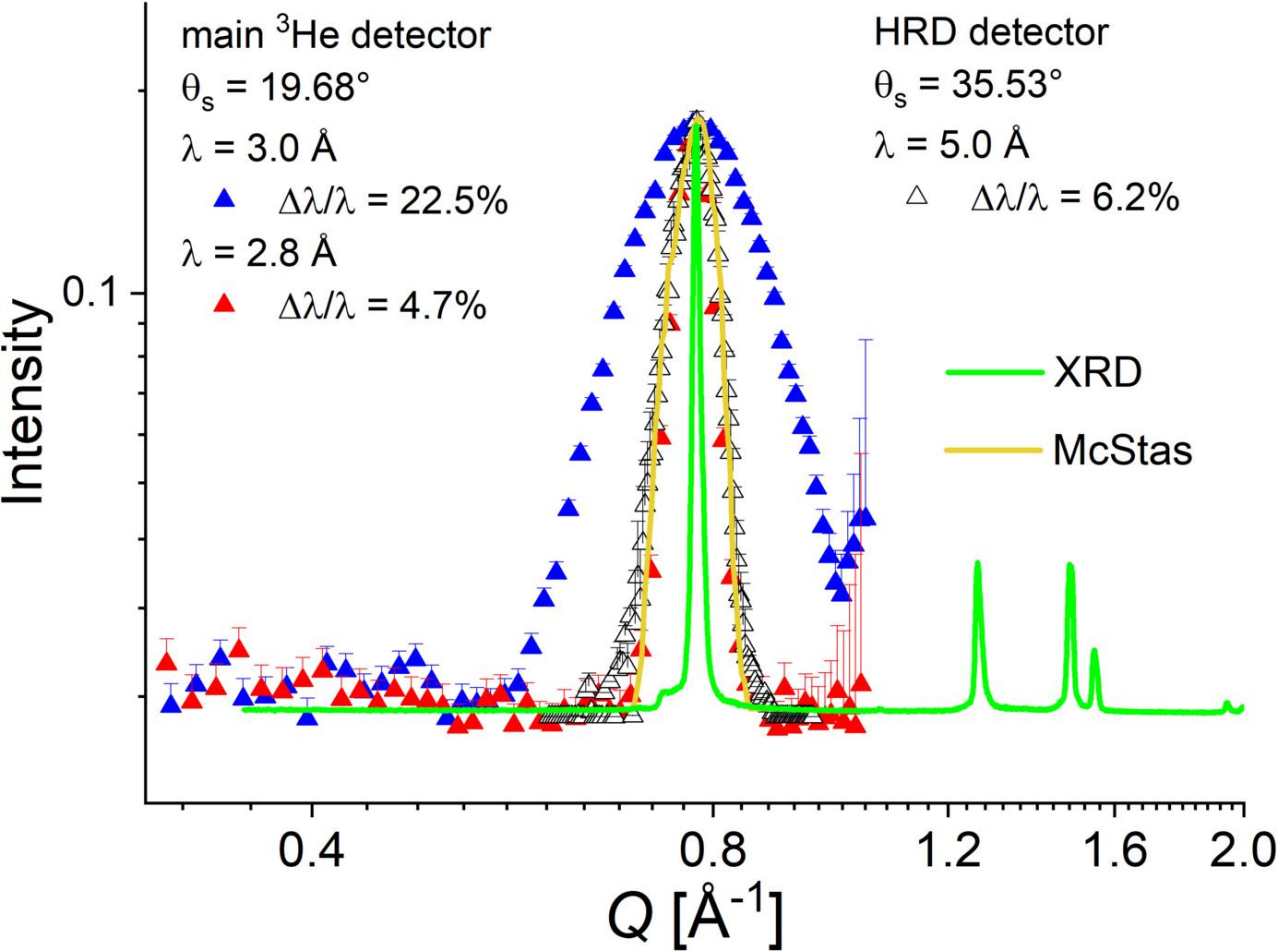
Measured (symbols) and simulated (yellow curve) one-dimensional scattering patterns from the fullerene-C60 powder sample under different experimental conditions in SANS and WANS geometries, as explained in the legends, in parallel with the XRD pattern (green curve). For better comparison, the XRD data were normalized to the SANS peak intensity and elevated so that the baseline corresponds to the flat SANS background.

**Figure 6:**
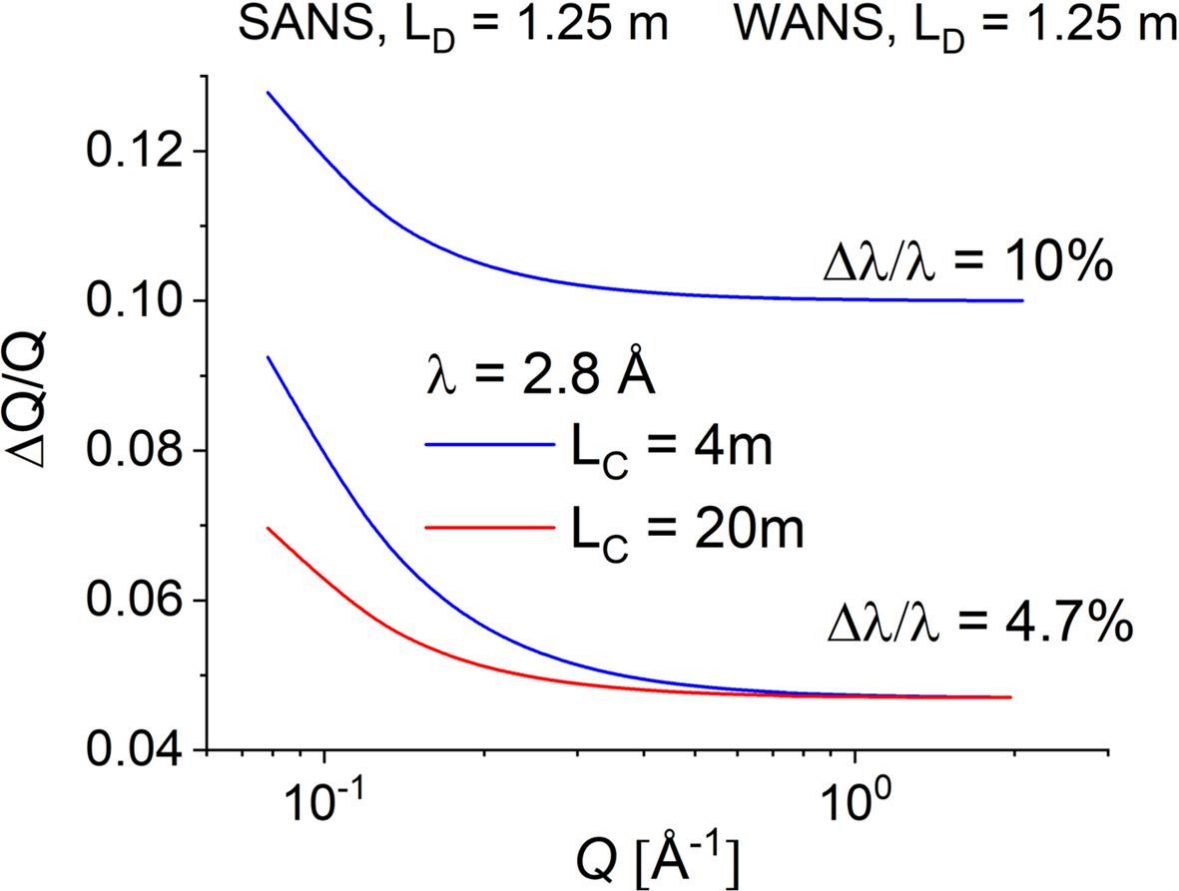
The *Q* resolution at KWS-2 for the SANS and WANS detectors under different experimental conditions (*L*_D_, *L*_C_, λ and Δλ).

The measurement of the 111 crystalline reflection of fullerene-C60 using the combination of SANS and WANS with different instrumental resolutions demonstrates the good quality of such results when also comparing the radially averaged 1D neutron scattering patterns with the XRD data (green line in Fig. 5 ▸). Moreover, the measured and simulated WANS signal from the fullerene-C60 powder sample (black open symbols and yellow curve in Fig. 5 ▸) coincide quite well for the same experimental conditions. This approach also proves that SANS and WANS measurements may be simultaneously carried out at a pinhole SANS diffractometer provided that the right resolution for different scientific goals (sharp crystalline peaks, form factor details, incoherent background determination *etc*.) may be selected in a versatile way. The instrument must also be equipped with wide-angle detectors that can operate in a fixed position simultaneously with the movable main detector, with an appropriate adjustment of collimation system. Finally, both kinds of detector should be able to collect data in TOF or continuous modes, depending on the scientific goals of the experiment. All this versatility is currently available at KWS-2, with the wavelength resolution tunable between 2 and 20% (Radulescu *et al.* 2015*b* ▸; Radulescu *et al.*, 2023 ▸). Thus we can reach the required resolution for resolving crystalline peaks, comparable to that of TOF-SANS instruments at spallation sources (Schiavone *et al.*, 2023 ▸). On the basis of the presented quality assessment of the WANS data, the WANS detectors [2D ^3^He tube arrays as reported by Radulescu *et al.* (2023 ▸)] are currently in the purchasing phase and the WANS option is in the planning phase at KWS-2. Finally, one should mention that in the *Q* range between 1 and 2 Å^−1^ the instrumental resolution is dependent on Δλ/λ with no observable effect from the collimation contribution (Fig. 6 ▸). This opens the possibility of improving the intensity on the sample by working with a short collimation *L*_C_ = 4 or 2 m in the intensity-unfavorable TOF mode, without a deterioration of the quality of the scattering features.

## 5. Conclusion

An analysis of the experimental data collected with tunable instrumental resolution over a wide angular range, beyond the limits of the conventional SANS setup, has been combined with *McStas* simulation results. The results demonstrate that the proposed WANS setup for the KWS-2 instrument will be suitable for measurement of fine scattering details up to θ_s_ = 50°. This will enable the targeted *Q*_max_ to be reached. It also raises the possibility of improving the signal-to-noise ratio by separating the inelastic scattering from the elastic scattering in TOF mode when data at high *Q* do not show a flat profile. A flight path of *L*_D_ > 1 m is needed to achieve this goal, as shown by Balacescu *et al.* (2021 ▸).
